# Molecular Mechanism of Vitamin D Receptor Modulating Wnt/β-catenin Signaling Pathway in Gastric Cancer

**DOI:** 10.7150/jca.81034

**Published:** 2023-10-02

**Authors:** Ying Zhang, Yan Li, Yuzheng Wei, Lei Cong

**Affiliations:** 1 Department of Radiotherapy, Cangzhou Central Hospital, Hebei, China.; 2 Department of Oncology, Shandong Provincial Hospital Affiliated to Shandong First Medical University, Jinan, China.; 3 Department of Oncology, Shandong Provincial Hospital, Cheeloo College of Medicine, Shandong University, Jinan, China.

**Keywords:** vitamin D receptor, gastric cancer, Wnt/β-catenin signaling pathway, FokI polymorphism, carcinogenesis

## Abstract

**Background:** Gastric cancer is the most common gastrointestinal cancer worldwide. The latest data showed that it was the fourth leading cause of cancer-related death. The unobvious symptom and the difficulties lying in the early diagnosis largely affect the effect of the treatment. Therefore, it becomes particularly important to investigate the related genes and signal transduction pathways in gastric cancer. Our previous study found that the vitamin D receptor (VDR) gene FokI polymorphism may be associated with susceptibility to gastric cancer in the Chinese Han population. However, the mechanism of VDR affecting gastric cancer is unknown. In this study, we explored the molecular mechanism and the possible signaling pathway of VDR modulating carcinogenesis and progression of gastric cancer.

**Methods:** The expression of VDR in gastric cancer cell lines was interfered by plasmid transfection and RNA interference technology. And then we analyzed the cell viability and invasive ability by MTT assay, colony formation assay, and transwell migration assay, and detected the expression of VDR and several signaling proteins in gastric cancer cells by SDS-PAGE and Western blotting.

**Results:** The overexpression of VDR can significantly inhibit the viability and invasive ability of gastric cancer cells; on the contrary, when VDR siRNA inhibits the expression of VDR, the viability and invasive ability of gastric cancer cells enhanced. VDR expression levels in gastric cancer cells treated with 1,25 (OH) 2D3 showed a time-dependent increased expression; and with the increase of the VDR expression, the expression of β-catenin decreased gradually, but the expression of E-cadherin showed a time-dependent increase (P < 0.05). Compared with the mutant-type VDR gene(ff) cells, the degree of β-catenin decline was significantly enhanced after transfected with homozygous wild-type VDR gene (FF) plasmids (p<0.05).

**Conclusions:** The results of this study indicate that VDR FokI polymorphism plays an important role in the malignant phenotype of gastric cancer cells, such as proliferation, invasion, and clone formation. When the VDR is activated by its ligand, it can prevent the nuclear import of β-catenin, affect the E-cadherin level, inhibit the proliferation of gastric cancer cells, which suggested that VDR FokI gene may play a role of cancer suppressor via Wnt/β-catenin signaling pathway.

## Introduction

Gastric cancer is the fifth most commonly diagnosed cancer and the fourth leading cause of cancer death worldwide^1^. It, following lung cancer, is the second most common type of cancer and the second leading cause of cancer-related deaths in China^2^. Early-onset gastric cancer incidence has increased in China in the past 30 years^3^. The cause of high mortality of gastric cancer mainly lies in early gastric cancer usually has no specific clinical manifestations. Most patients with newly diagnosed advanced gastric cancer lost the opportunity of surgical curative resection. Furthermore, gastric cancer is a multifactorial disease involved in the intricate web of signaling pathways and results from multiple exposures to environmental factors, lifestyle risk factors, and individual genetic predisposition^4^. This heterogeneity in gastric cancer remains a key obstacle in the development of targeted therapeutics and poses a great challenge for researchers. Thus, it is pivotal to identify the specific biomarkers of gastric cancer to develop treatments targeted to the specific tumor behavior.

Vitamin D receptor (VDR) is a member of the nuclear receptor family of transcription factors^5^. It can be activated by activation form of 1,25 (OH) 2D3. After binding to that ligand, VDR forms a heterodimer with another nuclear receptor RXR^6^. By identifying and binding to the specific binding sites in target gene regulatory sequence which called vitamin dresponse elements (VDREs), to regulate the transcription of the downstream target genes, producing a series of biological effects, such as cell proliferation suppression, apoptosis induction, cell signal disturbing, tumor suppression gene activity enhancing and oncogene expression suppression etc. 7, 8. Tumor related factors, for example tumor necrosis factor-alpha and RAS, are closely related to VDR expression^9^, ^10^.

**Figure 1:**
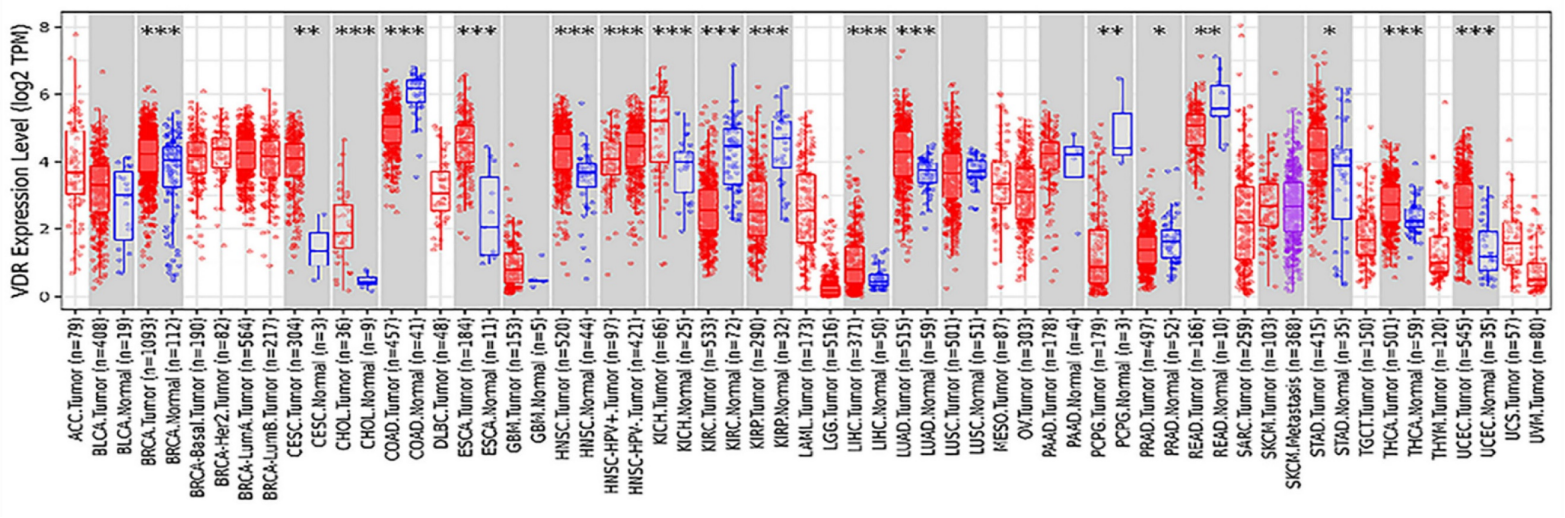
Expression of VDR in different tumor types from TIMER.

The polymorphism of VDR gene is significant. Most studies have focused on the FokI restriction enzyme sites which located in the transcription initiation site. This polymorphism can change the amino acid sequence length due to different translation initiation codon, eventually produce the same protein of different variants^11^, ^12^. VDR FokI f allele carriers have 3 more amino acids than F allele carriers^13^. F allele has lower transcription activity^14^. The FF genotype generally states the wild-type genotype because F is the more prevalent allele. The ff genotype states the mutant -type genotype. Previous studies have confirmed that the FokI polymorphism of VDR gene is associated with the incidence of prostate cancer^15^, breast cancer^16^, colorectal cancer^17^, ovarian cancer and skin cancer^18^, ^19^. A strong relationship between VDR polymorphism and gastric cancer predisposition was found in both Kashmiri and Iranian populations^20^, ^21^. It has also been reported that VDR FokI gene polymorphism is associated with Han and Uygur gastric cancer susceptibility in China^22^, ^23^. Miao et al.^24^ and Wen et al.^25^ demonstrated that VDR was lower expressed in gastric cancer tissues than in normal tissues. Later, Miao et al. proposed that the disease progression free survival and overall survival of VDR-negative patients were significantly shorter than those of VDR-positive gastric cancer patients^26^. However, according to the analysis of VDR expression in various tumors from the Tumor Immune Evaluation Resource database, we found that VDR was highly expressed in gastric cancer tissues and low expressed in adjacent tissues (Fig. 1). This contradiction may arise because of differences in research methods. In addition, the mechanism of VDR modulating the proliferation and invasion of gastric cancer is unknown.

In present study, we focus on the role of VDR in the proliferation and invasion of gastric cancer cells through cell experiments. At the same time, in order to explore the intracellular signal transduction pathway modulated by VDR in the development of gastric cancer. We explore the relationship between VDR and Wnt/β-catenin signaling pathway during the occurrence and development of gastric cancer.

## Materials & methods

**Cell cultures.** The human gastric cancer cell lines N87, MKN-28, MKN45, AGS and KATO III were provided by The Chinese Academy of Sciences cell bank. The five cell lines were cultured in RPMI 1640 Mediums supplemented with 10% fetal bovine serum and 1% Penicillin-Streptomycin Solution mixture at 37 °C in an environment containing 5% CO2.

**SDS-PAGE and Western Blotting.** Briefly, Protein samples were dissolved in a sample buffer containing 1mM β-mercapethnol. 40µg proteins samples were used to perform 12% SDS-PAGE (Solarbio) electrophoresis at 180 V for 60 min. Proteins were then transferred to a 0.45 µm cellulose nitrate strips membrane (GE Healthcare) at 4°C for 1h and then blocked for 1 h at room temperature in a solution of 5% (w/v) skimmed milk powder and 0.02% (w/v) Tween 20 in PBS, pH 7.5. It was incubated with appropriate primary antibodies (1:1000, CST) overnight at 4°C, and then washed with PBST and incubated with a horseradish peroxidase-labeled secondary antibody (1:5000, zhongshan) for 1h at room temperature. The detection was made with ECL (Pierce). The films were exposured by at different time points, and then developed and fixed.

**Transient transfection.** The cells were put at 80%-90% confluent density in 6-well plates, which were transiently transfected with 4 μg/ml DNA plasmids (including the overexpressed VDR plasmids, VDR wild-type and mutant-type plasmids, and empty vector plasmids) using Lipofectamine 2000 (Invitrogen) according to manufacturer's instructions. For each well to be transfected, dilute 10 μl Lipofectamine 2000 in 250 μl Opti-MEM (gibco) Medium without serum in a separate tube. Mix gently and incubate for 5 minutes at 37℃. Dilute 4 μg/ml DNA plasmids in 250 μl Opti-MEM Medium without serum in the separate tubes. Mix gently. After the 5 minutes incubation, add the diluted Lipofectamine 2000 to the tubes with the diluted DNA molecules. Mix gently and incubate for 20 minutes at room temperature to allow complex formation to occur. The solution may appear cloudy, but this will not impede the transfection. Add the solution containing DNA molecule-Lipofectamine™ 2000 complexes to each well. Rocking the plate back and forth. Incubate the cells at 37°C in a CO2 incubator for further experiments.

Cells were plated and cultured in growth media until cell density reached to 30-50% prior to siRNA transfection using Lipofectamine 2000. siRNA sequences are as following: 5'-GGAAGAAUGUGGAGCUCAATT-3'. Dilute 20pmol siRNA oligomer in 50ul Opti-MEM with low-serum medium, and dilute 1ul Lipofectamine2000 in 50ul Opti-MEM with low-serum medium. The above diluted siRNA oligomer and diluted Lipofectamine2000 form siRNA-Lipofectamine 2000 complexes. Add the siRNA-Lipofectamine^TM^ 2000 complexes obtained in the previous step to each hole containing cells and culture medium, and gently shake the culture plate back and forth to mix fully. The cells were cultured in 5%CO2 and 37 ℃ incubator for 24-96 hours, and the culture medium was changed every 6 hours. The effect of gene knockout was measured for follow-up test.

**Cell function assay.** MTT assay was tested to study the reproductivity and metabolic activity of human gastric cancer cell lines. The above three groups of cells were selected to adjust the cell concentration to 2 x 10^4^ / ml, and were inoculated on 96-well culture plates. + VDR hole, -VDR hole, control hole and zero hole were set up. After the cells were cultured in 5%CO2 and 37 ℃ incubator, 20ul of MTT was added to each well and cultured for 4 hours. The OD value was determined at the wavelength of 490nm by enzyme labeling instrument.

Cell viability was analyzed by colony formation assay. Briefly, ~ 700 cells were added to each well of a 6-well culture plate. After 2 weeks of incubation, cell colonies were washed twice with PBS, fixed with 4% para -formaldehyde for 15 min and then stained with 0.5% crystal violet for 30 min. The excess stained was removed. Individual clones with ≥ 30 cells were counted.

Cell invasive and migration ability were analyzed by transwell assay. Selected the BioCoat Matrigel invasion chamber of BD company, refered to the manufacturer's instructions, added 0.5 ml cell suspension (about 5 x 104 cells number) in each 24 pores plate chamber. Culture 22 hours in the incubator, carefully wipe the non-invasive cell above the film using cotton swab. Small room were fixed with 100% methanol, stained with crystallization violet. Counted the invasive cells under the microscope.

## Results

### The protein expression of VDR in gastric carcinoma cell lines

**Figure 2:**
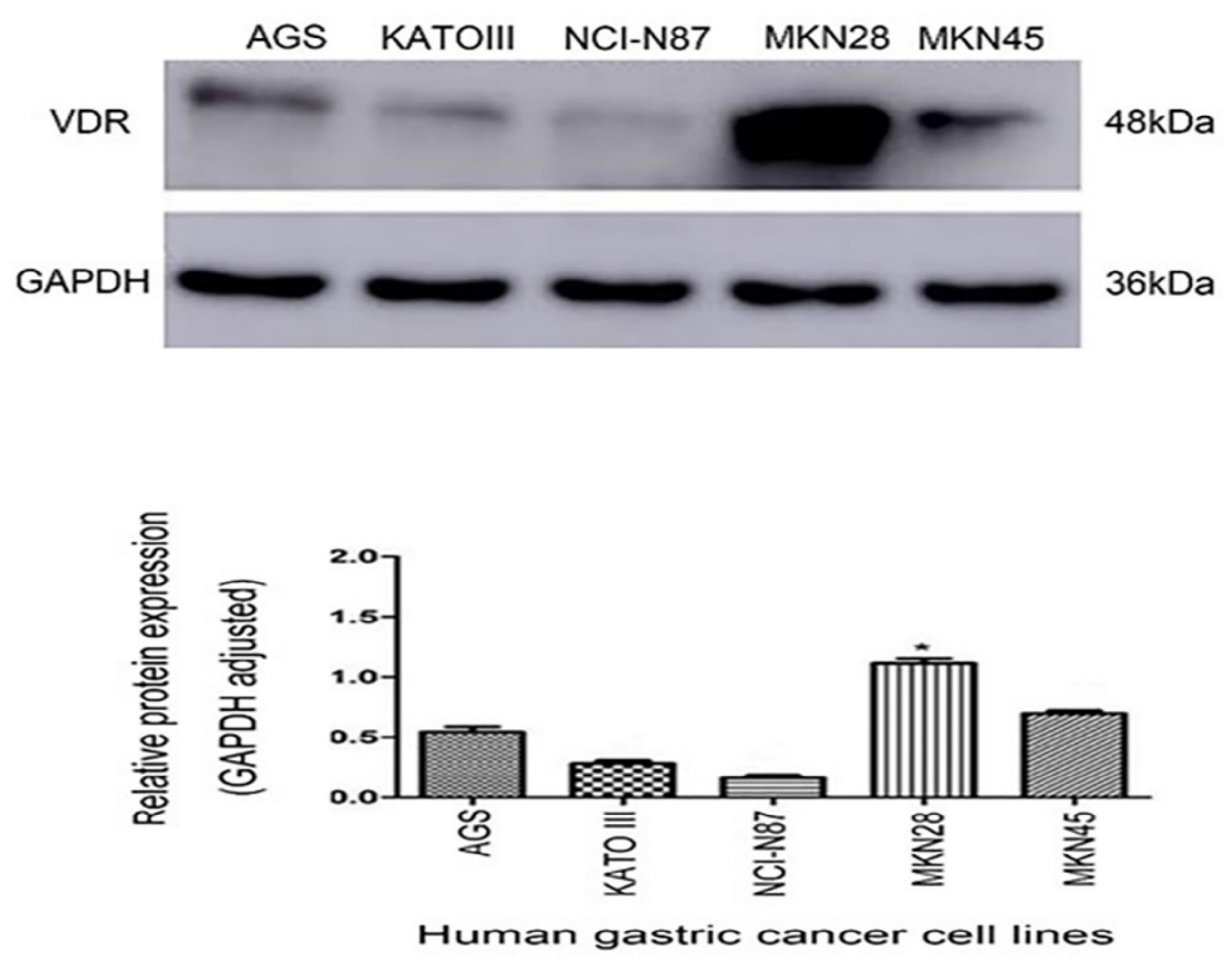
Expression of VDR in five gastric carcinoma cell lines.

There are VDR expression in gastric carcinoma cell lines AGS, KATO III, N87, MKN-28 and MKN45. VDR expression was significantly reduced in cell line N87, whereas was the highest in cell line MKN-28. (Fig. 2). To avoid the influence of the VDR genotype of the cell line itself on the subsequent results, we selected the N87 cell line with the lowest VDR expression.

### Function changes of gastric cancer cells induced by interference on expression of VDR

**Figure 3:**
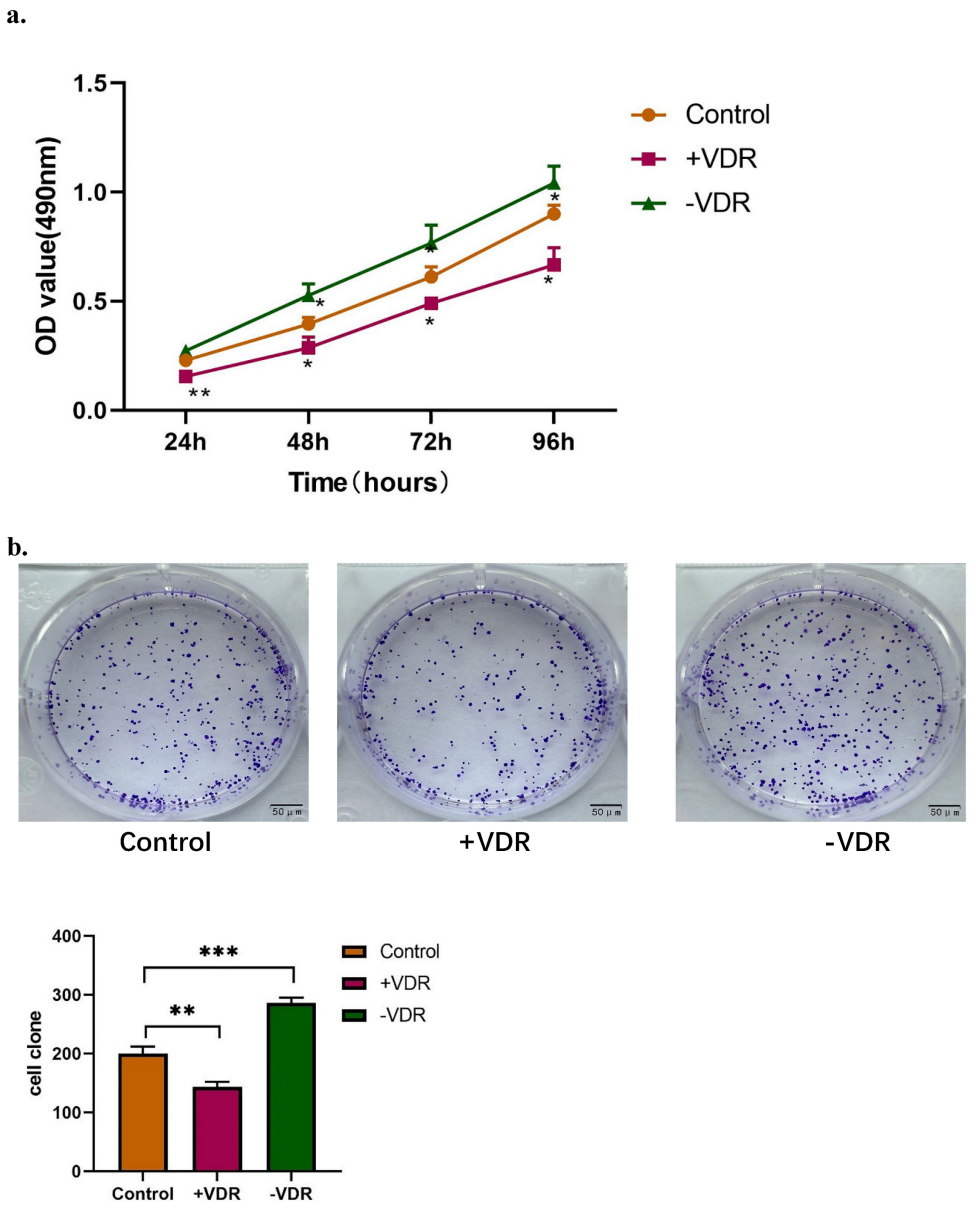

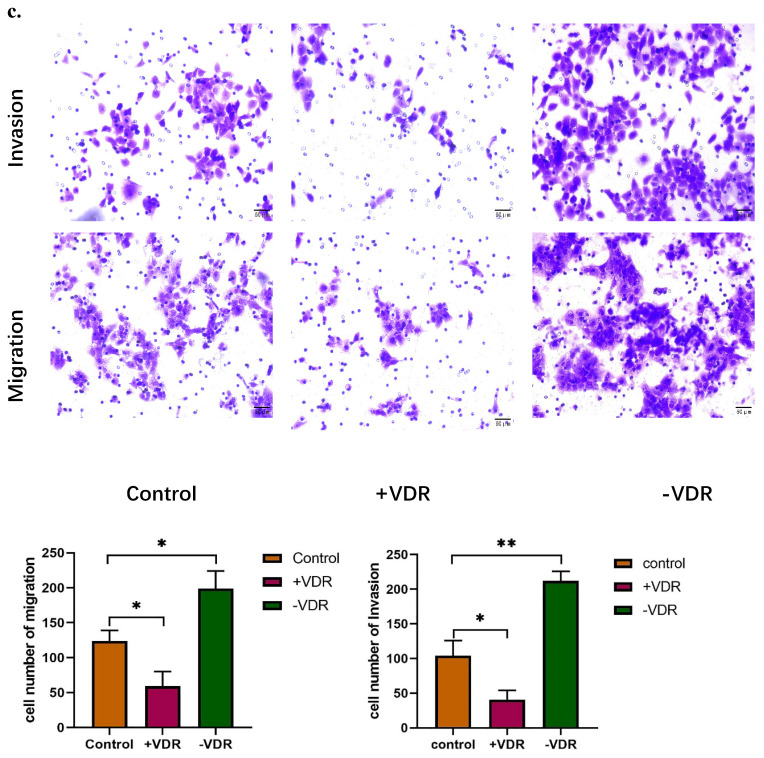
Function changes of gastric cancer cells induced by interference on expression of VDR. **a.** Cell viability affected by VDR were analyzed by MTT assay. **b.** Cell proliferation capability affected by VDR were analyzed by colony formation assay. **c.** Cell invasive and migration ability affected by VDR were analyzed.

The expression of VDR in gastric cancer cell line was interfered by plasmid transfection and RNA interference technology. Cell viability and invasive ability were analyzed by MTT assay, colony formation assay and transwell migration assay. In this experiment, there are one control group and two experimental groups: the N87 cell line (control group), which natural state of VDR expression was the lowest; the N87 cell line transfected with VDR plasmid (+VDR group) and the N87 cell line transfected with SiRNA plasmid (-VDR group). The data in Fig. 3a show that when VDR plasmid was transfected into N87 cell line in +VDR group, the overexpression of VDR can significantly inhibit the growth of cells, on the contrary, when VDR siRNA inhibits the expression of VDR in -VDR group, the cell grows rapidly.

The colony formation assay showed that the number of clones formed in the +VDR group was significantly decreased; however, the number of clones formed in the -VDR group was significantly increased (Fig. 3b).

When SiRNA plasmid were transfected into gastric cancer cell in -VDR group, its invasive and migration activity increased significantly, on the contrary, cells in +VDR group decreased the invasive and migration activity (Fig. 3c).

### The links between VDR and Wnt/β-catenin signaling pathway

**Figure 4:**
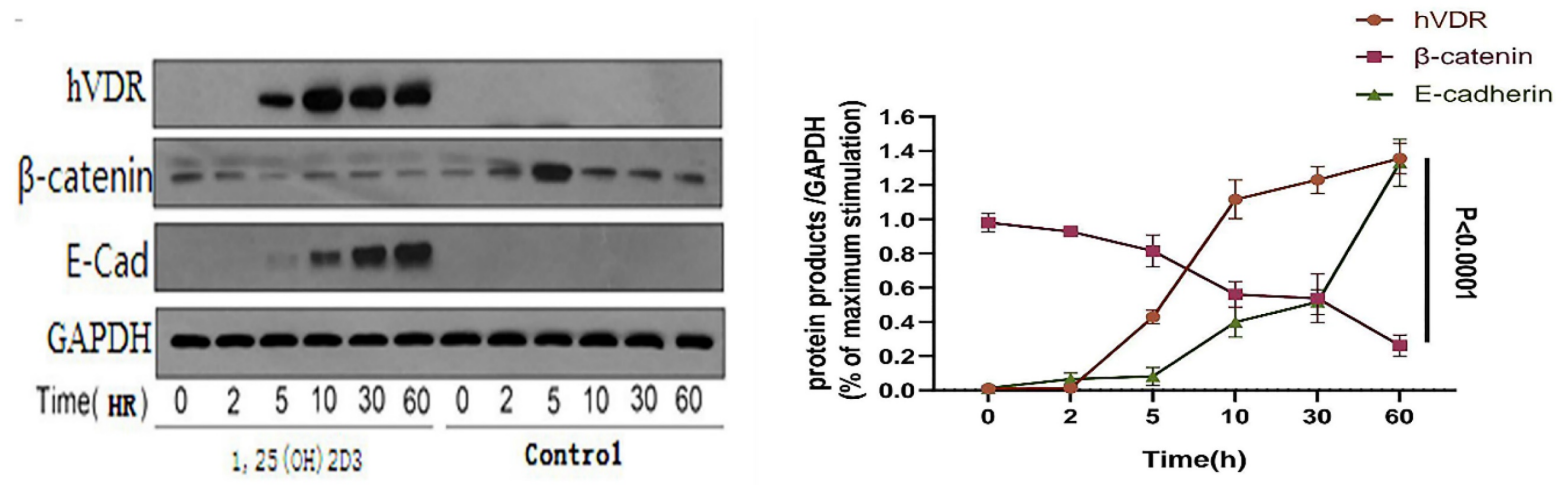
Changes of VDR and related signaling proteins after 1,25 (OH) 2D3 stimulation.

VDR was stimulated with activated 1,25 (OH) 2D3 in gastric cancer cells, using ethanol (final concentration 0.1%, it doesn't affect the living status of cells) as a control group. Then we detected the level of β-catenin, c-myc protein and E-cadherin by western blot analysis, to clear the links between VDR and Wnt/β-catenin signaling pathway. Gastric cancer cell line (N87) was treated with 1,25 (OH) 2D3 for 0, 2, 5, 10, 30, 60 hours respectively. Western blot indicated that VDR expression levels in gastric cancer cells treated with 1,25 (OH) 2D3 showed a time dependent increased expression; and with the increase of the VDR expression, the expression of β-catenin decreased gradually, but the expression of E-cadherin showed a time dependent increase (P < 0.05) (Fig. 4).

**Figure 5:**
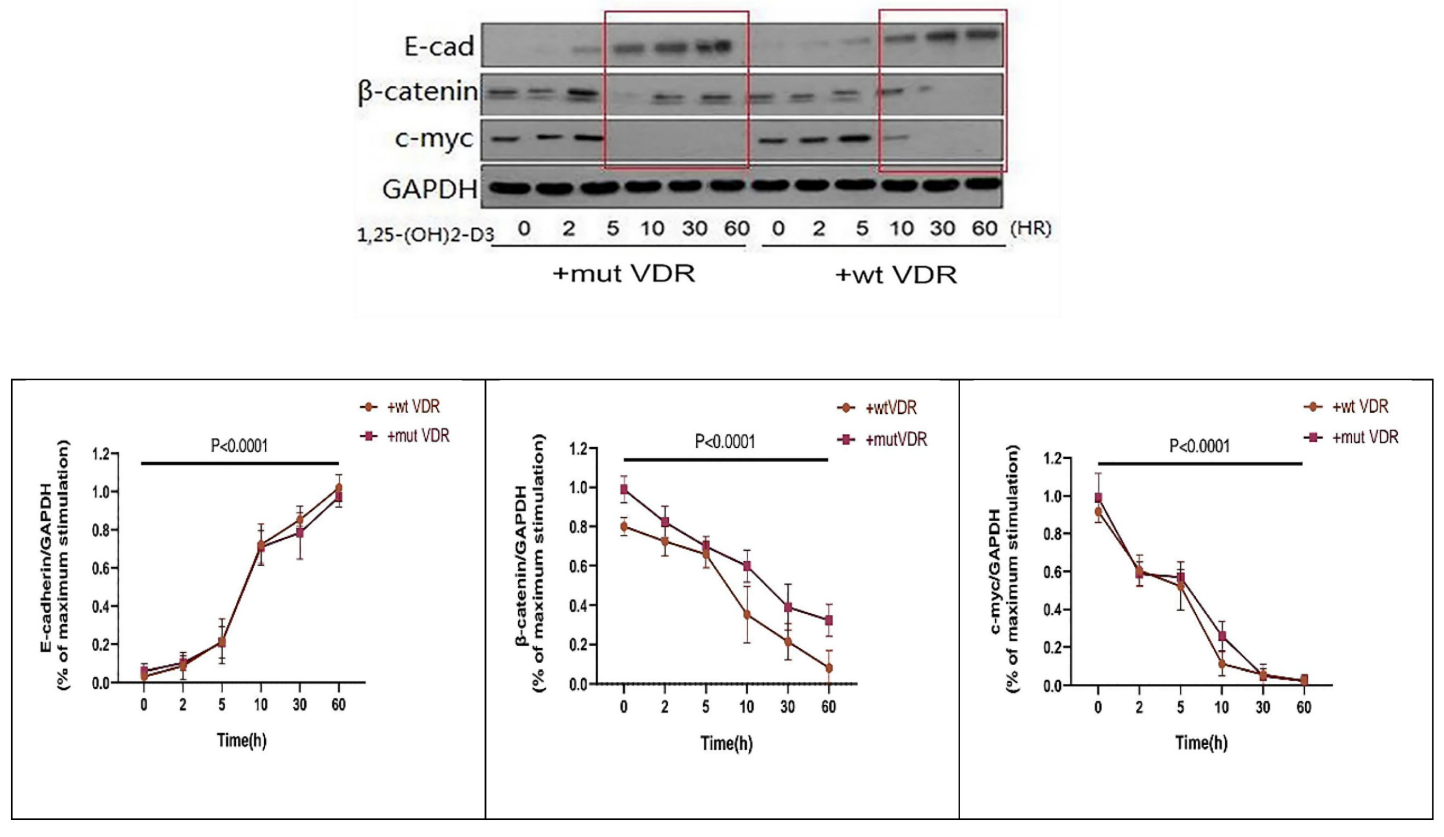
Variation of Wnt/β-catenin pathway signal proteins in different VDR genotypes with ligand stimulation.

In order to observe the links between VDR and Wnt/β-catenin signaling pathway in gastric cancer cells with different VDR genotypes, we transfect the wild-type and mutant-type VDR expression plasmids into gastric cancer cells by Lipofectamine2000 respectively. The gastric cancer cell line N87 was transfected with wild-type and mutant-type VDR gene plasmids, and were treated with 1,25 (OH) 2D3 for 0, 2, 5, 10, 30, 60 hours respectively. As shown in Fig. 5, in wild-type group (+wt VDR) and mutant-type group (+mut VDR), the expression of β-catenin and c-myc protein were decreased especially after a long time treatment, but the expression of E-cadherin in the cytoplasm increased over time. Compared with the mutant-type VDR gene cells (+mut VDR), the degree and the time of decline of β-catenin protein expression were significantly enhanced after transfected with wild-type VDR gene plasmids (+wt VDR) (p<0.05).

### Expression of β-catenin and cell proliferation in different VDR status cells

**Figure 6:**
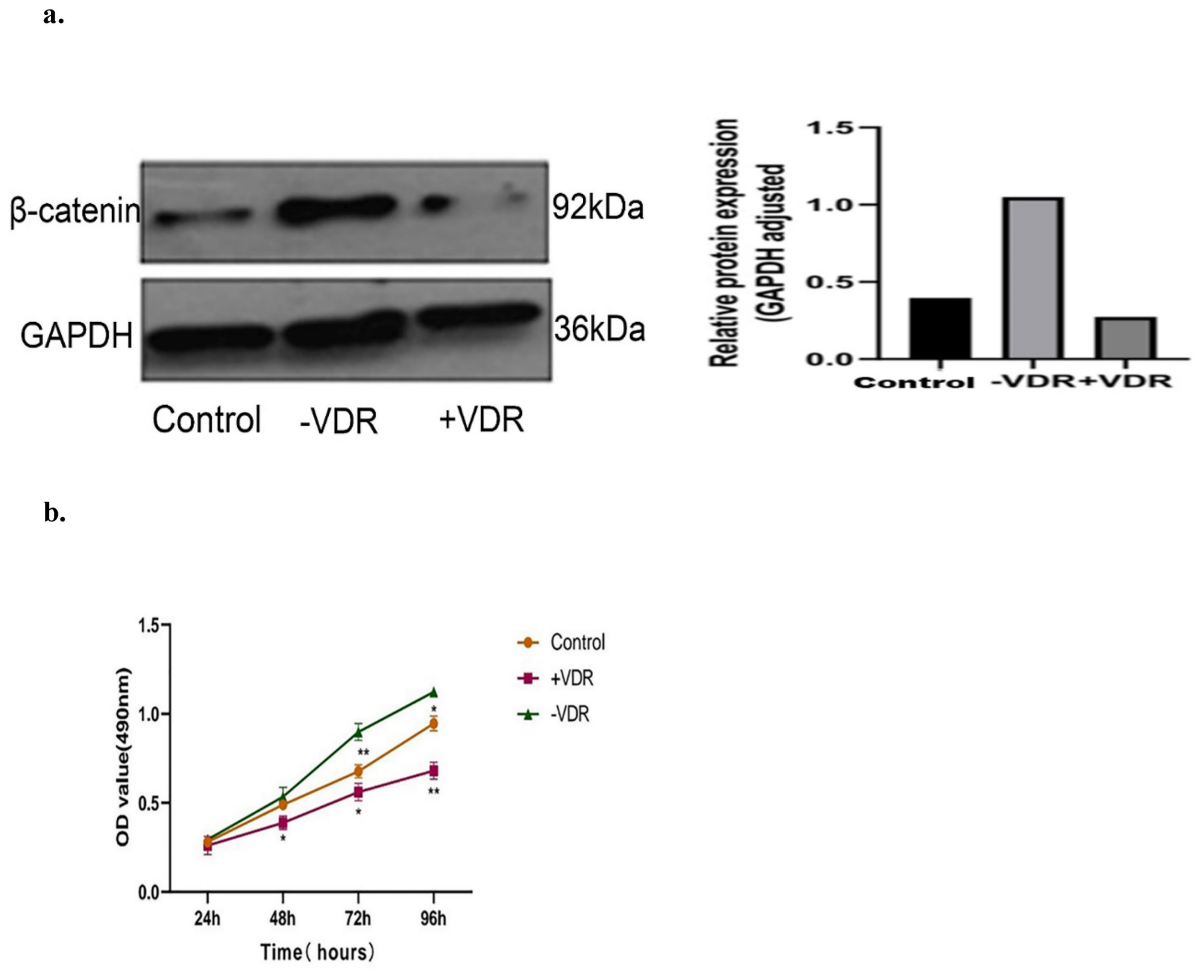
Effects of different VDR states on β-catenin expression and cell proliferation. **a.** Effects of different VDR states on β-catenin expression. **b.** Effects of different VDR states on cell proliferation.

There are one control group and two experimental groups in this experiment: the N87 cell line (control group), the VDR plasmid transfected cell (+VDR group) and the SiRNA transfected cell (-VDR group). We detected the level of β-catenin by western blot analysis, and analyzed the cell viability by MTT assay. As presented in the Fig. 6a, increasing the expression of VDR in gastric cancer cells in +VDR group, the expression of β-catenin decreased; on the contrary, the expression of β-catenin increased. As presented in the Fig. 6b, increasing the expression of VDR in gastric cancer cells in +VDR group, the cell proliferation was significantly inhibited; on the contrary, cell proliferation was enhanced in -VDR group (p<0.05).

## Discussion

Genomic and proteomic screening methods have demonstrated that VDR is a crucial determinant of tumor cell response to 1,25 (OH) 2D3 27. The VDR gene also modulates a variety of independent biological processes in cancer^28^. Existing evidence supports that VDR can inhibit the proliferation, invasion and metastasis of tumor cells. In this study, we found that VDR can affect the malignant phenotype of gastric cancer cells, such as proliferation, clone formation, invasion and migration. Compared with the cells transfected with mutant-type VDR gene plasmids, the decrease of β-catenin and c-myc protein expression after transfection of VDR Fok I wild plasmids were significantly enhanced and rapid, which suggested that VDR FokI gene can play a role of cancer suppressor via Wnt/β-catenin signaling pathway.

Helicobacter pylori infection remains a major cause of gastric cancer 29. VDR has an antimicrobial activity against Helicobacter pylori 30, 31. The VDR signaling pathway can promote c-Raf/MEK/ERK phosphorylation and prevent apoptosis in Helicobacter pylori-infected GES-1 cells^32^. In addition, one study reported that VDR expression was significantly lower in gastric cancer tissues, and that among cancer tissues VDR was higher expressed in well differentiated tissues and in small tumors^25^. Together these observations demonstrated that VDR was a protective factor. In present study, we further confirmed correlations between VDR and malignant phenotype of gastric cancer in the N87 cell lines. VDR impinged on proliferation, clone formation, invasion and migration in the N87 cell lines. Similar results have been reported in other papers. 1,25 (OH)2D3 or vitamin D analogues can produce significant antitumor effects by regulating proliferation, apoptosis and angiogenesis through VDR and can be used to treat gastric cancer 33. A study showed that vitamin D analogue EB1089 induced apoptosis in gastric cancer cells through the VDR and mitochondrial apoptosis pathways^34^. 1,25 (OH) 2D3 induced miR145 through VDR to inhibit colony formation, cell viability and induce cell arrest at S-phase by targeting E2F3 and CDK6 in gastric cancer^35^. These results demonstrated that VDR may be a prognostic factor for gastric cancer. Wnt signaling pathway is a highly conserved signaling pathway. If the key protein in Wnt signaling pathway mutations, resulting in abnormal signal activation, it is possible to induce the occurrence of cancer and improve the ability of cancer cells to evade the immune system^36^, ^37^. In the presence of Wnt binding, Dishevelled (DVL) is activated. Activated DVL is part of a protein complex that recruits GSK-3 away from the degradation complex, allowing the dephosphorylation and nuclear import of β-catenin and subsequent gene induction via binding to the T cell factor/lymphoid enhancer factor (TCF/LEF)^38^.

Previous studies have shown that Wnt/β-catenin signaling pathway was an important factor in the occurrence and development of many solid tumors^39^-^44^. In gastric cancer cells, Wnt/β-catenin pathway is involved in epithelial-mesenchymal transition (EMT). Reduced Wnt/ β-catenin signal can prevent the occurrence of EMT, inhibiting the the invasion and metastasis of gastric cancer^44^-^46^. In human colon cancer cells, 1,25 (OH)2D3 exerted antitumor effects through VDR induction of E-cadherin and inhibition of β-catenin signaling^47^. In view of the important role Wnt pathway had played in malignant tumor formation process, we have reason to believe that Wnt/β-catenin signaling pathway is one of the mechanisms that VDR contributes to the development of gastric cancer. The results presented in this article indicate that the VDR expression increased, the β-catenin expression in nucleus accordingly reduced, and E-cadherin expression showed a corresponding increase in gastric cancer cells stimulated by 1,25 (OH)2D3. β-catenin accumulates in the nucleus and start the transcription of downstream target genes such as c-myc, MMPs, and cyclin D1, which leads to the occurrence of abnormal cell proliferation and tumor^48^, ^49^. It indicates that when the VDR is activated by its ligand, it can induce a series of reactions to prevent the nuclear import of β-catenin, thereby inhibiting the proliferation of gastric cancer cells, and regulating the invasion and migration of gastric cancer cells via affecting E-cadherin level. Increasing the expression of VDR in gastric cancer cells, the expression of β-catenin decreased, while the cell proliferation was significantly inhibited; on the contrary, the expression of β-catenin increased, and cell proliferation was enhanced with the decrease of VDR expression, which further confirmed the effect of VDR on the proliferation and apoptosis of gastric carcinoma cells by regulating the level of β-catenin.

VDR FokI has been shown to influence the translation initiation position of 1,25 (OH)2D3 and thus its downstream effects^50^. Uitterlinden et al. 50 suggested that some promoter regions of vitamin D target genes may be more sensitive to this VDR F allele -dependent difference in activity. A meta-analysis found a strong association between VDR FokI with poorer overall and progression-free survival in Lung cancer^51^. Alimirah et al. 52 have found that VDR f allele may play a role in amplifying aggressive breast cancer. Similarly, consistent with our results, compared with mutant-type VDR gene cells (+mut VDR), the degree and the time of decline of β-catenin and c-myc protein expression were significantly enhanced after transfected with wild-type VDR gene plasmids (+wt VDR), while the cell proliferation was significantly inhibited. We further strengthen the conclusion that the VDR FF genotype may instigate a more intense response to preventing gastric cancer than VDR ff genotype counterpart. Such a result also validated our previous conclusion that the patients with f allele (Ff and ff genotype) were associated with a poorer histological differentiation and a higher level of CRP, indicating a more severe inflammation condition and worse prognosis of gastric cancer^22^.

In conclusion, our study further researched the relationship of VDR and Wnt/β-catenin signaling pathway and the function mechanism in evolution process of gastric cancer. We also provided evidence for distinct functional differences between VDR ff genotype and VDR FF genotype genetic variants in gastric cancer cells, in expect to providing clues for the follow-up studies. The VDR FF genotype may play a greater role in the reduction of gastric cancer, paving the way for understanding why some gastric cancer cells do not respond effectively to vitamin D treatment. We hope the frame elaborated in this research can be regarded as a reference for the early diagnosis, prognosis estimation and targeted therapy in gastric cancer.

## Abbreviations

VDR: Vitamin D receptor
VDREs: vitamin dresponse elements
DVL: dishevelled
TCF/LEF: T cell factor/lymphoid enhancer factor
EMT: epithelial-mesenchymal transition
